# Toward Learning Machines at a Mother and Baby Unit

**DOI:** 10.3389/fpsyg.2020.567310

**Published:** 2020-11-13

**Authors:** Magnus Boman, Johnny Downs, Abubakrelsedik Karali, Susan Pawlby

**Affiliations:** ^1^Department of Software and Computer Systems, School of Electrical Engineering and Computer Science, KTH Royal Institute of Technology, Stockholm, Sweden; ^2^Child & Adolescent Psychiatry, Psychological Medicine and Integrated Care, Clinical Academic Group, The National Institute for Health Research Maudsley Biomedical Research Centre, King's College London, London, United Kingdom; ^3^NVIDIA Corporation, London, United Kingdom; ^4^Channi Kumar Mother and Baby Unit, Bethlem Royal Hospital, South London and Maudsley National Health Service Trust, London, United Kingdom

**Keywords:** learning machine, machine learning, multi-modal learning, mental health, maternal unresponsiveness, mind-mindedness

## Abstract

Agnostic analyses of unique video material from a Mother and Baby Unit were carried out to investigate the usefulness of such analyses to the unit. The goal was to improve outcomes: the health of mothers and their babies. The method was to implement a learning machine that becomes more useful over time and over task. A feasible set-up is here described, with the purpose of producing intelligible and useful results to healthcare professionals at the unit by means of a vision processing pipeline, grouped together with multi-modal capabilities of handling annotations and audio. Algorithmic bias turned out to be an obstacle that could only partly be handled by modern pipelines for automated feature analysis. The professional use of complex quantitative scoring for various mental health-related assessments further complicated the automation of laborious tasks. Activities during the MBU stay had previously been shown to decrease psychiatric symptoms across diagnostic groups. The implementation and first set of experiments on a learning machine for the unit produced the first steps toward explaining why this is so, in turn enabling decision support to staff about what to do more and what to do less of.

## 1. Introduction

An annotated data set of videos from the Channi Kumar Mother and Baby Unit (MBU) of Bethlem hospital in Southern England was analyzed by means of Machine Learning (ML). The purpose was to assist the in-patient psychiatric MBU researchers, who benefit from more than 30 years of video analysis experience and video feedback interventions already, to understand if and how ML could assist them in their work. The automatic analysis of mother-baby interaction could support the goal to improve outcomes. All mothers admitted to the 13-bedded MBU are offered video feedback sessions in the first week of admission and after their final discharge meeting. Baby cooperativeness, less maternal unresponsiveness and passiveness in their babies, and improved maternal sensitivity are among the outcomes strived for (cf. Stephenson et al., 2018, in which the MBU is also described in detail). Ideally, data-driven research by ML experts could complement the picture of how to best improve outcomes, including early interventions for the baby, which might prove important later in life (Cannon et al., 2002; Zeegers et al., 2019). Data scientists in this way complement an already multidisciplinary team of psychiatrists, psychologists, nurses, occupational therapists, social workers, and nursery nurses.

In-patient MBUs provide the opportunity of supporting mothers with severe mental illness in developing their relationships with their infants via individualized video feedback intervention. In earlier studies (Kenny et al., 2013), exposure control groups for measured changes included a community-based ill group of mothers with a mental health diagnosis of similar severity, living at home and without the intervention (negative control), and a group of healthy mothers. The MBU mothers and their infants showed improvements in their interactions, measured via responsiveness and infant cooperativeness. Mothers, upon discharge from the MBU were significantly more sensitive, cooperative, and responsive than the negative control group, and as attuned as the healthy group. What remains to be studied is what part the video feedback intervention has played to establish these excellent results, and here the learning machine is central.

The conception that any video recording of human-to-human interaction contains hidden patterns that may be revealed by ML methods, thereby improving the chances of a human observer to understand what is really going on in the interaction, has lots to recommend it (Martinez et al., 2017). There is evidence for the fact that human micro-expressions undetectable to the human eyes of the observer carry information, for example, even in videos with only a single human object (Pfister et al., 2011). If a mother and her baby were placed in a situation staged for cameras to capture micro-expressions, such analyses could be carried out. While the situation at the Channi Kumar MBU is staged to facilitate analyses, however, there were no high-resolution cameras in place. To detect and decode human micro-expressions in the face, high frame rates are required, and filming is typically done in a studio fitted with an expensive camera rig. Here, the filming was done with simpler cameras, historically to VHS tapes and recently to digital media, and panning and scanning or similar technical techniques to zoom in on where the action is when it comes to human expression were not used. This should come as no surprise, as the material was gathered over several decades, and the goals of the research were different (Kenny et al., 2013). Features useful to supervised machine learning approaches include metadata for a video, annotations that indicate when in time important events occur in the video, phenotypical information about the mothers as obtainable from their health records, and natural language processing analyses of in-video speech. The latter are currently in the planning stages, and will include negation detection and sentiment, stylometrics, and semantic embeddings.

More evidence for hidden pattern detection is provided by successful projects employing deep learning (Li and Deng, 2020). Through a layered representation of what is on the videos, such systems can over time learn to classify new videos, based on the responsiveness of the mother, for example. The problem is that deep learning requires very large amounts of data to train and test on. In the case of this MBU, there are hundreds of videos, but not millions. One could argue that there are millions of videos somewhere in the world depicting mother-baby interactions, and that training and testing could be done by somehow employing this video material, using transfer learning (cf. Gebru et al., 2017). But in this MBU, the cameras were set up to capture specific situations, and neither the mothers nor their babies are typical. Their mental condition and the situation they are placed in differs so much from the situation in which most mother-baby videos are recorded that classical deep approaches can not be used. For these reasons, relational neural networks (Santoro et al., 2017) is an example of how relatively data-poor domains can benefit from machine learning in ways that are more feasible for the problem at hand.

Babies are also particularly problematic for ML-based vision processors. They tend to cover all or parts of their face with waving hands or a toy, or completely turn away from the camera. Their faces are typically chubby, making some expressions extra hard to detect and classify for a machine. Naturally, the language used by babies as captured on tape also obfuscates the baby's true intent or desire; if a machine were to listen to it, it would at least at the outset have a tremendous disadvantage compared to a human expert on infant interaction. What an ML-system tries to learn from the MBU videos could in philosophical terms be described as *mimesis*: an imitation game involving various forms of desire. Plato's term for the rest of a story was *diegesis*: a narrative. At the MBU, diegesis must come from sensemaking: whatever the machine does to the video material needs to be related to the clinical practice and critically accepted by the stakeholders as a means to positive change (Boman and Sanches, 2015). A video is thus a simplified sample from a diegetic process—a para-linguistic snippet of a complex reality—which the machine analyses through a mimetic algorithmic process. The results are then fed back to researchers at the MBU, who can appropriate and place the mimetic results into the larger narrative, viz. the world that has meaning to the patient and the clinician. That world has lots of contextual information that the machine has not. For example, mothers have been assigned ICD-10 diagnoses and have had their predominant clinical symptoms assessed by a psychiatrist (Stephenson et al., 2018).

A *learning machine* can be defined as an autonomous self-regulating open reasoning system that actively learns in a decentralized manner, over multiple domains (Boman et al., 2018). Its purpose is usually not to replace, but to augment, humans in its logical or physical vicinity. With repeated training, testing, and use, a correctly programmed learning machine will increase its usefulness over time and over task (Boman et al., 2019). The same data point can over time contribute to many rounds of perception and reasoning in the machine. Crucial to success is the data representation: how to engineer the features in order to facilitate ML, a process referred to as *representation learning* (Bengio et al., 2013). Each video will be classified based on representations of the mother-baby interaction. More specifically, the classification will be done by binning the video material in classes where similar videos appear together, based on the features employed by the machine. Simpler regression tasks that may be realized by standard ML-methods will also be carried out, as part of a process of understanding what the learning machine can achieve when run in self-supervised mode.

In order to investigate the feasibility of implementing a full learning machine for the MBU, a pilot study was carried out in which an ML pipeline was implemented for a small number of videos. The balanced accuracy, precision, and recall measures were computed and the identification of a signal of association were found. Because the pilot study was exploratory and merely served as a test of feasibility, the quantitative results are not presented in this paper. Instead, the normalized gain of the fully implemented system will be computed in order to make possible for the clinicians to assess the collected contributions of the ML pipeline, in terms that make sense to them in their daily work.

## 2. Materials and Methods

Evaluating the long-term effects of perinatal mental health intervention at MBUs is hard, which has led to a focus on patient preference and process rather than outcome (Stephenson et al., 2018). In the present study, learning machines represent a long-term focus on outcome. Human annotators at the MBU use coding systems and quantitative scores for various dimensions in mothers and babies. Similar scores have been used for several decades (see e.g., Pfister et al., 2011) for face recognition, emotion recognition in the human face, gait analysis, and for analysing various incongruences in human behavior. The by far most used score is the Facial Action Coding System (FACS) (Ekman and Friesen, 1978). The FACS score has been used to analyse the risk of repeated suicide attempts in depressed people (Archinard et al., 2000). In that small study, four human annotators, all extensively trained in FACS scoring, blindly scored repeaters (*n* = 11) and non-repeaters videotaped answering the single question “Do you think that 1 day, you will commit suicide?” Because of the training required and because each video takes a long time to code—Ekman himself says 6 months and an hour per 1-min video (Ekman, 1982)—means to automating scoring have been investigated in many domains. The two computer scientists in the here reported research have previously used the FACS for understanding spontaneously expressed emotions in the human face, and much of their experiences were reused for the MBU work.

### 2.1. Data

The MBU data is multi-modal—there is text, video, and audio—and amenable to data-driven processing (cf. Atrey et al., 2010). The text comes in the form of annotations made by the human observer, documented using pen and paper, and placed in a filing cabinet. The interactions were also transcribed verbatim (Pawlby et al., 2010). For this project, the cabinet contents were sampled during a site visit, to get a feel for its usefulness for validation of ML-based research. It was decided there and then to let the learning machine attempt to interpret the video by computing outputs that at least to some extent overlap with the human annotations. Since the human annotations are not guiding the learning strategy, the learning machine is agnostic, with respect to the expert knowledge that the MBU researchers possess. Hence, the machine makes no *a priori* assumptions about which class a new instance belongs to, but instead is an unbiased generalizer (Mitchell, 1980). A long-term goal would be to let the machine annotate each video in order to provide an agnostic form of note-taking (corresponding to mimesis). These pairs of notes could then be compared to investigate if the human annotator was biased by background knowledge of the mother and baby (corresponding to diegesis). Such comparisons would go far beyond bias and confounders, however, effectively functioning as a validation tool that could be used, e.g., to achieve benchmarked basic results (Ramirez et al., 2011) and then over time and task learn how to improve these results further (cf. Rendell et al., 1987).

The videos were all made when the baby was as near as possible mid-way between feeds and not sleeping. In other words, when mother and baby came to make the video, the baby was alert. The audio is of good quality but since no baby is older than 15 months, it consists mostly of the mother talking her baby with the intent of getting the baby interested in something, like a toy. Most of the dialog is thus semiotic, rather than oral, but may still constitute an important part of a multi-modal analysis via ML-based audio processing (Eyben et al., 2015; Laukka et al., 2016). There are videos in which the psychologist comes into the room in which the mother and her baby have been carefully placed for filming. In these videos, there is a limited amount of dialog between mother and psychologist.

For a first set of experiments, a sample of 136 videos was made available to the ML experts, in the form of digitalized files. The featurizer OpenFace (Baltrusaitis et al., 2016) was trained on two datasets: Labeled Face Parts in the Wild (LFPW) (Belhumeur et al., 2013) and Helen (Le et al., 2012), and the CLNF patch experts on the Multi-PIE (Gross et al., 2008), LFPW, and Helen training sets. A second set of experiments is currently in preparation with more than 1,500 videos, all of which have been pre-processed for data-driven exploratory research. A secure graphics processing unit has been equipped with the CUDA parallel programming platform for this purpose. There are five distinct kinds of technical challenges associated with the video data:

- Contrast
- Camera set-up
- Resolution
- Inactivity
- Length of each video.

In the camera industry, improved contrast came only with customer requests from forestry looking at wood types (mahogany vs. redwood, etc.) and from food industry (dark chocolate vs. milk chocolate, etc.). Contrast became an issue for Kodak and other companies developing film technology (Vox, 2015). In the videos, poor lighting is the cause for most ML pipeline failures. Note that a video might be usefully interpreted even with failures. Some videos are filmed from the back of the mother, others using mirrors. In the ones with mirrors, the mother is sometimes leaning forward, leading to the back of her head obstructing the view of the baby's face. There are also videos of mother and baby sitting together on a carpet full of toys. These are harder to use because they both look down on the carpet (where the toys are). There are even videos where the baby's face is upside down. Even if the pipeline is rotation invariant in theory, an upside down face does produce an extra challenge. Regarding resolution, the sample has videos from older batches that are problematic because they lead to more failures due to lower frame rate. Some videos are only a minute long, giving very little room for failure. Sometimes items from the above list of challenges combine. There are videos of carpet play only minute-long, for example.

The sensitive nature of the video material makes it impossible to share, except for a few mothers who explicitly gave permission to do so, but all findings (including bugs) regarding the open source pipeline used will be openly reported on. In addition, abstract facial features and landmarks can be anonymized and will be made available.

### 2.2. Ethics

Written informed consent was obtained from the individuals for the publication of any potentially identifiable images or data included in this article. The study of mother-baby interactions was approved by the Institute of Psychiatry, King's College London Ethics of Research Committee (reference 05/Q0706/159). Informed written consent was obtained for the recordings of the interactions to be used for research, and all procedures were conducted in accordance with the British Psychological Society ethical guidelines.

### 2.3. Previous Research at the MBU

Previous research at the MBU has been hypothesis-driven and quantitative, and has investigated the quality of mother-baby interaction, with healthy controls (Pawlby et al., 2010; Kenny et al., 2013). Video-taped play sessions have been analyzed by developmental psychologists and nurses using the CARE-Index, a screening tool to assess the quality of adult-infant interaction in general (Crittenden, 2004). Conclusions were based on about five minutes of unstructured playful interaction that occurs under non-threatening conditions in a room at the Channi Kumar MBU, and results from feedback sessions. The CARE-Index assesses the relationship between mother and baby, focusing on seven aspects. Four aspects focus on affect (facial expression, verbal expression, affection, and body contact) and three on temporal contingencies (turn-taking, control, and developmental appropriateness of chosen activity). Each aspect is evaluated and summed into scale scores. The scores for adults are sensitivity, unresponsiveness, and controlling. For the infant there is cooperativeness, difficultness, compulsivity, and passivity. In addition, more qualitative research used the concept of *mind-mindedness* (Meins, 2013): the mother's ability to read her baby's internal states, ascribing a mind to her baby. These research strands were linked by hypothesizing that mothers with severe mental illness (*n* = 50) would score lower than healthy mothers (*n* = 49) (Pawlby et al., 2010).

Four dimensions of behavior were coded in the mothers:

- response to change in infant's direction of gaze,
- trying to get the baby's attention,
- pause in the interaction, longer than 3 s,
- touching the baby,

with scores expressed as frequencies. Two dimensions of baby behavior were coded:

- change in gaze direction,
- gazing to the mother,

again using frequency scores.

A recently published study at the MBU (Stephenson et al., 2018) paired two more quantitative maternal scores. The Brief Psychiatric Rating Scale (BPRS) scores (Leucht et al., 2005) and the Health of the Nation Outcome Scales (HoNOS) (Wing et al., 1998) were also collected at admission and discharge. The changes in scores were calculated and binned into

- marked improvement,
- minimal improvement,
- no improvement, or decline.

The mind-mindedness was assessed from video taped on admission to hospital (pre-intervention) and compared to video taped on discharge (post-intervention). Such assessments are of special interest here since ML-based methods can make similar analyses, which could then be compared to the human ones with respect to results and observations. The supervised learning will be dictated by the labels used by the clinic for their manual assessments and annotations. In addition, the phenotypical information on the mother, e.g., demographics or age, will be added to the data used for training the learning machine. This leads to several prediction tasks for the machine, ranging from simple binary classifications (e.g., responsive mother vs. non-responsive mother) to regression tasks relating to the seven scores employed in the manual assessments of mothers and their babies (see Kenny et al., 2013). With the human labels and scores as a gold standard, adherence to this standard will be measured for 1,500+ videos in a larger future study, by means of sensitivity and specificity. With features set up as the relevant Action Units, the supervised machine learning problems will not only produce quantitative results related to the gold standard, but the relative feature importance will also be analyzed, via PCA and SHAP. Besides Action Units, the pipeline also yields landmarks, head orientation, and gaze. Extra features will be calculated based on these measures, in the form of second-order measures, like duration of eye contact.

### 2.4. Learning Machine Research Methodology

The new ML-based research was, by contrast, hypothesis-less and data-driven. This choice was based on earlier experience of using the FACS for spontaneous emotion detection in videotaped sequences. The vision processing started from the OpenFace (Baltrusaitis et al., 2016) implementation for head orientation, landmarks, and gaze. Rather than starting from scratch, multi-modal fusion techniques developed by others (Corneanu et al., 2016) were adapted for use. Feature extraction was not done in reference to a particular hypothesis or model. Instead, conditional local neural networks were trained on each part of the face, with the locality pertaining to a particular face region. This training allowed for landmark detection and eye gaze estimation. Head orientation was then computed from the relative positions of the landmarks, given a 3D-landmarks template. This approach was chosen for its scalability to massive data sets, paving the way for quite general artificial intelligence applications (Bengio and LeCun, 2007).

Facial landmark detection is a key element in analysing facial expressions and one of the most promising and robust approaches is the Constrained Local Neural Field (CLNF) (Baltrusaitis et al., 2013). It is an instance of the Constrained Local Model (CLM) that deals with the issues of landmark detection in complex scenes. In general, there are three main parts to a CLM: a point distribution model, patch experts, and the fitting approach used. The first part models the location of facial landmarks in the image using non-rigid shape and rigid global transformation parameters. Therefore, this part learns the spatial relationships between facial parts' features. For example, mouth landmarks are below the nose landmarks, and eyes are to each side of the nose. The patch expert learns the appearance of local patches around landmarks of interest: what each facial part looks like. The last part of the CLM is the fitting approach in which the best fit of the features point on the patch is found.

OpenFace represents state-of-the-art for landmark detection. For the point distribution model and the fitting approach, the most common approach is iteratively searching for the best fit using Regularized Landmark Mean Shift (Saragih et al., 2011). For the second part of the CLM, a so-called local neural field was used as patch expert. This is an instance of conditional random fields that include a neural network layer between the input and output layers. Therefore, it takes advantage of the non-linearity representable by neural networks together with the flexibility of the conditional random fields. Three instances of the detector were used, one for facial landmark detection, and one for each eye to detect the gaze.

## 3. Results

To automatically extract FACS-codes and let these guide the features in the learning model has a long history (Bartlett et al., 2005). However, it is only in recent years that ML has been used for classification and regression tasks in such efficient ways that hopes have been raised for automating annotation. A learning machine trained on general features for human-human interaction can now bin specific and previously unseen examples into classes of similar videos. If the learning is self-supervised, the machine may or may not come up with exactly the same classes recognized by human experts. This opens up for learning machine tasks of prediction for the MBU data. For example, a regression task for the machine could be to project new examples on to the categories of the CARE-index, or on to Cohen's criteria of small, medium, or large effect (Cohen, 2013).

**Figure 1:**
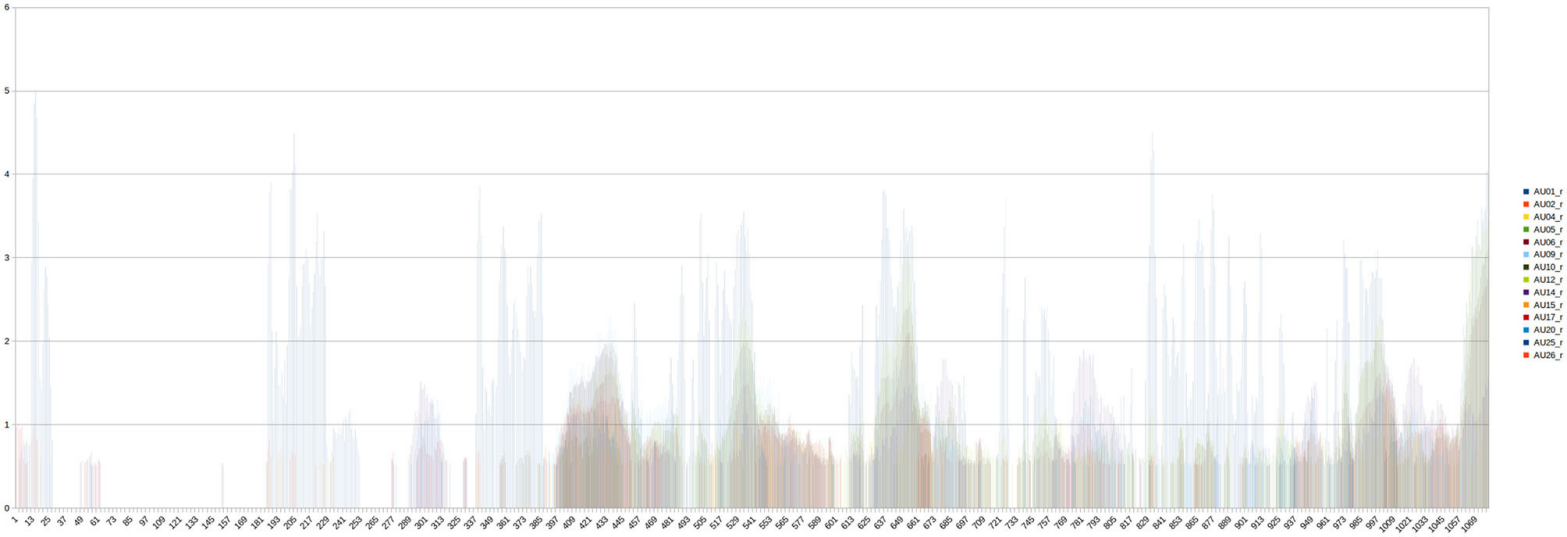
Example of how to pedagogically illustrate the intensity of Action Units, for an example video from a different domain sample, with FACS-codes related to embarrassment. The challenge to grasp the semantics from this plot is considerable.

Quantitative scores can be kept in flat structures like tables, or plotted, e.g., over time elapsed in the video. To human observers, even for experts, it can be hard to grasp the semantics of quantitative scores (Figure 1). A much better way is to superimpose the features on to the original video (Baltrusaitis et al., 2018). For the MBU data and the CARE-Index, an approach very similar to what was used for spontaneous emotion data sets and the FACS was adopted. The approach is exploratory, because the learning machine starts out agnostically, and only then gets validated by comparisons to human expert annotations and classifications. To avoid overfitting and increase its resilience, the learning machine uses random elements (e.g., from training on noisy signals) and relies on random sampling to jump to conclusions about characteristics of populations (e.g., by shuffling its training data for every epoch). In order to meet scientific criteria for reproducibility, all random seeds are stored.

When it comes to learning from video, the general problem of how to utilize context is extremely important. Since video is just sequences of still images, a special case is that of frames depicting an unfolding of an emotion expression, a change in gaze, or some other relevant human expression. The size of the sliding window, i.e., how many frames to employ to capture the unfolding, is one thing that requires experimentation. This could even pertain to a lookahead, for predicting evolution of the expression at hand. How to measure the Δ, i.e., the change from frame to frame, is another question that has been approached using data-driven methods. Only experiments show how to optimize settings for best results, given the data set at hand, and therefore the work needs to be open-ended and explorative. Computational complexity is not so interesting from a theoretical point of view, because of its focus on the worst case, but for good scientific reporting algorithmic analysis is needed to look at average case complexity and the computational complexity of the canonical model (Purdom and Brown, 1995).

**Figure 2:**
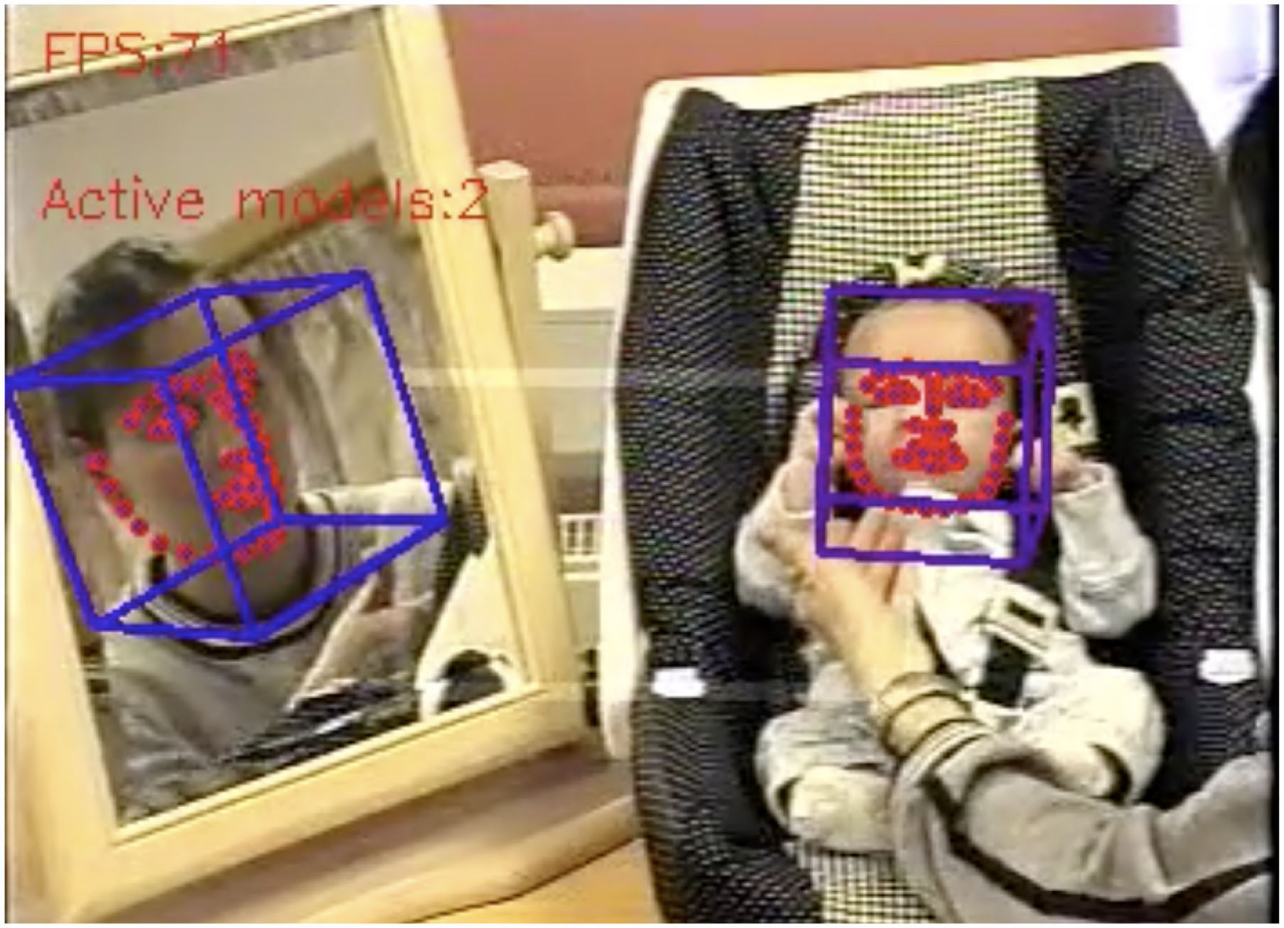
With the mother's face captured in a mirror, the ML pipeline is able to recognize her face and associated features. Her arm and parts of her head are visible at right. The baby is in this camera set-up fixed in a chair facing the camera, resulting in good recognition, even if hands and arms of both baby and mother occasionally gets in the way.

Using the ML pipeline described in the previous section, resting on the most recent version of OpenFace (Baltrusaitis et al., 2018), it proved possible to superimpose information on the input videos from the sample, resulting in output videos with face recognition, gaze, and orientation (Figure 2). In each output video, failures result in the overlays disappearing for a short time, which is usually notable but the analysis is usually still helpful.

Besides a general understanding of what kinds of model are adequate for the MBU, it is useful to outline experiments along the lines of what has already been published based on manual observations and annotations, using ordinary statistics. Setting up for regression from automatic video analyses into parameter value ranges for mothers and babies is in some cases straightforward while in other cases extensive data pre-processing is required. Rather than trying to outperform some qualitative output score, the emphasis here is on investigating the usefulness of manually obtained results for partial automation. The validation of ML-experiments is likewise simple, trying to achieve a reasonable baseline given the gold standard provided by human researcher efforts.

A large number of ML-methods could perform the regression task. As the experiments are ran modulo a canonical model rather than in the context of trying to maximize the value of some particular well-defined goal function, classical parts of training, testing, and validating ML-methods will here be left out. Hence, there is but little hyperparameter optimization, boosting, or factorization at this stage (cf. e.g., Halko et al., 2011).

**Figure 3:**
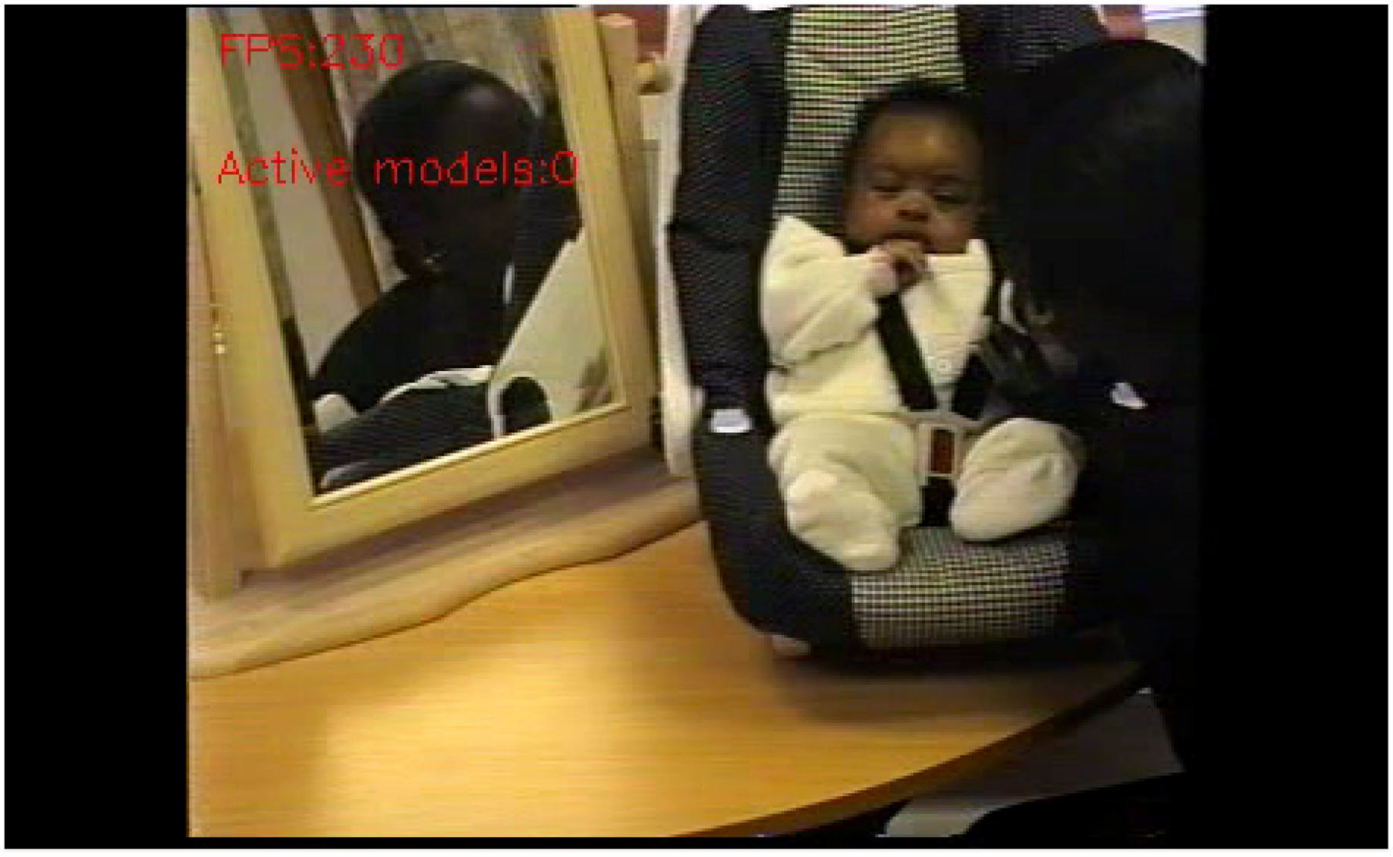
The faces of mother and baby remain unrecognized after minutes, leading to serious vision parsing failure.

**Figure 4:**
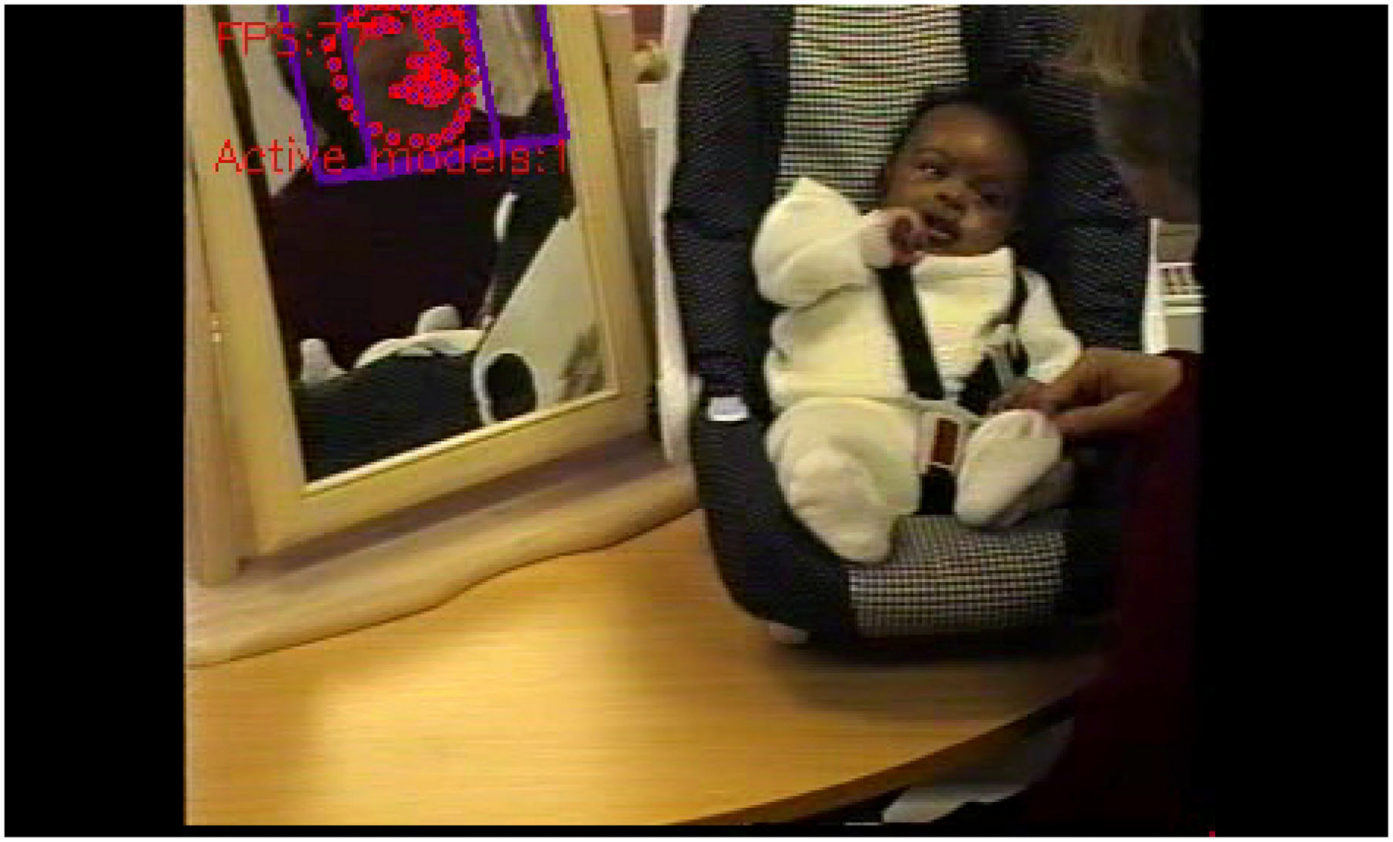
The researcher's face is recognized within seconds, without failure. The baby's face is recognized only after a long time, and with repeated short failures.

Bias is what makes ML-methods viable. Still, unwanted bias in training data may cause problems for those same methods. In the pipeline for the MBU data, a flagrant example was the insufficient training examples for non-white faces of mothers and babies. This led to algorithmic bias which in turn caused the machine's inability to recognize a dark-skinned mother as well as her baby (Figure 3). When a white researcher came into the room, the machine immediately picked up on her face (Figure 4). There is no easy way to resolve this problem, which is well-known among ML researchers, but less known among social scientists (Zou and Schiebinger, 2018). Since the approach here is agnostic, much of the ML-method employment is totally data-driven and hence reliant on the skewed data. Only in the next step, the sensemaking, which involves human reflection and a critical stance toward algorithmic results can this and similar embarrassments be handled.

## 4. Discussion

Previous research has found the MBU stay to decrease psychiatric symptoms across diagnostic groups (Stephenson et al., 2018). What ML could assist with is understanding at an even deeper level why this is so. Results from initial experiments show that such further analyses are possible.

The intense manual labor at the Mother and Baby Unit on annotations and scoring was contrasted with a possible automation of analyses of video material capturing mothers and their babies, as well as their respective annotations. Usefulness to the staff at the unit was the main criterion when designing, implementing, and testing a learning machine for this purpose. A pipeline to feed input to the machine was tested on a sample of 136 videos. The testing details were not given in this paper, as the main object was feasibility—including barriers to successful implementation, such as algorithmic bias—at this stage. Next steps are to run the pipeline adding ten times as many videos, to train the learning machine on related data sets for transfer learning, and to fuse signals from three different modalities (text, audio, video) with almost disjoint feature sets. The machine learning methods employed will vary with the requirement of each specialized classifier for each modality. This is essentially a mixture of experts approach, with each classifier acting as a weak uni-modal expert. With the pipeline in place, multi-modal fusion for the efficient classification of videos can be achieved, so that automated data-driven and agnostic clustering of behaviors can be realized. The actionable insights gained from such classifications constitute an important milestone for making learning machines serve the staff at the unit in a practically useful and meaningful way.

## Data Availability Statement

The data analyzed in this study is subject to the following licenses/restrictions: the datasets used for this study are sensitive but can be further described at meta-level to researchers upon request. Some mothers gave their consent to showcase them extensively, and the examples shown here are pertaining to members of that group of mothers. Requests to access these datasets should be directed to Susan Pawlby, susan.pawlby@kcl.ac.uk.

## Ethics Statement

The study of mother-baby interactions was approved by the Institute of Psychiatry, King's College London Ethics of Research Committee (reference 05/Q0706/159). All procedures were conducted in accordance with the British Psychological Society ethical guidelines. The patients/participants provided their written informed consent to participate in this study. Written informed consent was obtained from the individuals, and individuals' next of kin, for the publication of any potentially identifiable images or data included in this article.

## Author Contributions

SP led the clinical work. JD led the data pre-processing work. AK led the machine learning. MB wrote up the paper, with contributions from all authors, and together with AK designed the learning machine.

## Conflict of Interest

The authors declare that the research was conducted in the absence of any commercial or financial relationships that could be construed as a potential conflict of interest.
